# The Unwearied Action of the Heart

**Published:** 1870-02

**Authors:** 


THE UNWEARIED ACTION OF THE HEART.—The effect of every thing that touches the heart is multiplied by the intensity of the heart's own changes. Hence it is that it is so sensitive, so true and quick an index of the body's state. Hence, also, it is that it never wearies. Let me remind you of the work done by our hearts in a day. A man's total outward work, his whole effect upon the world in twenty-four hours, has been reckoned about three hundred and fifty foot-tons. That may be taken as a good "hard day's work." During the same time, the heart has been working at the rate of one hundred and twenty foot-tons. That is to say, if all the pulses of a day and night could be concentrated and welded into one great throb, that throb would be enough to throw a ton of iron one hundred and twenty feet into the air. And yet the heart is never weary. Many of us are tired after but feeble labors; few of us can hold a poker out at arm's length, without, after a few minutes, dropping it. But a healthy heart, and many an unsound heart, too—though sometimes you can tell in the evening, by its stroke, that it has been thrown off its balance by the turmoils and worries of life—goes on beating through the night when we are asleep, and when we wake in the morning, we find it at work, fresh as if it had only just began to beat. It does this because upon each stroke of work there follows a period, a brief but a real period of rest; because the next stroke which comes is but the natural sequence of that rest, and made to match it; because, in fact, each beat is, in force, in scope, in character, in every thing, the simple expression of the heart's own energy and state.—*Appleton's Journal.*

